# Advocating for both Environmental and Clinical Approaches to Control Human Strongyloidiasis

**DOI:** 10.3390/pathogens5040059

**Published:** 2016-09-30

**Authors:** Meruyert Beknazarova, Harriet Whiley, Kirstin Ross

**Affiliations:** School of the Environment, Flinders University, GPO Box 2100, Adelaide 5001, Australia; harriet.whiley@flinders.edu.au (H.W.); kirstin.ross@flinders.edu.au (K.R.)

**Keywords:** strongyloidiasis, *Strongyloides stercoralis*, anthelminthic drugs, nematicides, ivermectin, resistance

## Abstract

Strongyloidiasis is an underestimated disease caused by the soil-transmitted parasite of the genus *Strongyloides*. It is prevalent in socioeconomically disadvantaged communities and it is estimated that global infection could be as high as 370 million people. This paper explores current methods of strongyloidiasis treatment, which rely on administration of anthelminthic drugs. However these drugs cannot prevent reinfection and drug resistance has already been observed in veterinary models. This highlights the need for a combined approach for controlling *Strongyloides* that includes both clinical treatment and environmental control methods. Currently, nematicides are widely used to control plant parasites. The review suggests that due to the species’ similarity and similar modes of action, these nematicides could also be used to control animal and human parasitic nematodes in the environment.

## 1. Introduction

Strongyloidiasis is a disease caused by two soil-transmitted helminths of the genus *Strongyloides*, *Strongyloides stercoralis* and to a lesser extent *Strongyloides fuelleborni*. *S. fuelleborni* is only found in Africa and the Southeast Asian countries of Papua New Guinea, while *S. stercoralis* is globally distributed and is clinically more important [1,2]. Strongyloidiasis is an underestimated disease highly prevalent in socioeconomic disadvantage communities [3,4]. Although it is currently estimated that about 30–100 million people are infected globally, a more accurate estimate is thought to be around 300 million [2,5] or up to 370 million people [6]. Due to the ability of *S. stercoralis* to remain in a host organism as autoinfective filariform larvae (L3), a person can stay asymptomatically infected for decades [7,8]. Parthenogenesis allows a single remaining female parasite present in a host to reproduce and cause reinfection, which has serious ramifications for effective treatment [1,9,10]. The survival of asexually produced infective larvae (L3) is estimated to be less than 14 days. However, heterogonically developed infective larvae (L3) have been shown to survive indefinitely in the soil of optimal environment conditions until they find a host [9,11,12]. The complicated *S. stercoralis* life-cycle, insensitivity of detection methods and social factors challenge strongyloidiasis identification, diagnosis and treatment [3,13]. If not diagnosed in time it can lead to fatal outcomes [1,14], and given that we see high disease rates in population sub-groups, strongyloidiasis is not only a personal but a public health issue [2,15].

*Strongyloides* genus species have both parasitic and free-living life cycles. The infection of a human starts from infective larvae (filariform larvae L3), which penetrate the host and are transported via blood to the lungs, from where the larvae migrate to the gastrointestinal tract. In the intestine, larvae moult two times to become adult female worms, which hatch eggs through parthenogenesis and produce rhabditiform larvae. The rhabditiform larvae can either be excreted in feces or become infective filariform larvae autoinfecting a host [7]. Certain respiratory conditions; however, are believed to affect the filariform larvae transition through the lungs and cause its development into adult egg laying female worms in the lungs [16,17]. The repeated cycle of this leads to pulmonary strongyloidiasis [18]. There are no further studies done showing the filariform larvae maturing into adult worm in the lungs. The pulmonary strongyloidiasis is believed to occur due to autoinfection and filariform larvae disseminating to respiratory system [19]. Currently, the nematode’s environmental stage has not been extensively studied or controlled. However, exploring mechanisms to control the nematode in the environment should be made a priority. Biocontrol has been described as a promising way of environmental control of agricultural, animal and human soil-transmitted nematodes [20], and the use of commercially available nematicides should be considered and explored. 

At the World Health Organization global parasite control meeting in 2004 it was recommended that *S. stercoralis* control measures should be included in the health package for endemic areas [21]. However, to date, there has been no progress made mostly due to the gaps in knowledge regarding *S. stercoralis* treatment and control [22]. Investigation into transmission hot-spots is currently being undertaken [23]. To address the transmission, the best management approaches need to be identified and this discussion represents a step in this process. For example, wastewater overflow in septic tanks, solid waste including diapers or other animal feces might be areas to target.

This paper reviews currently commercially available drugs used to treat human strongyloidiasis, and explores the main issues associated with drug application. In addition, this paper looks at nematicides registered in Australia, their use, main constituents, mode of action and toxic effects. To date, strongyloidiasis treatment has tended to be viewed only from a clinical perspective, which is an inevitable part of treatment once infection has occurred. However, drug treatment cannot prevent reinfection and there is the potential for drug resistance. Here we suggest a combined approach of strongyloidiasis treatment; through clinical intervention with drugs once infection has occurred, but supplemented with nematode control in the environment. The advantages and concerns with both approaches are discussed.

## 2. Anthelminthic Drugs

The World Health Organization currently recommends albendazole and ivermectin as suitable drugs against strongyloidiasis. Mebendazole is not recommended anymore, as it has been demonstrated to have a suboptimal effect against strongyloidiasis (Table 1) [24]. Ivermectin has been shown to be the most effective and therefore the first choice drug in strongyloidiasis treatment [25], especially for chronic strongyloidiasis [26,27].

### 2.1. Benzimidazoles (Albendazole and Mebendazole)

Benzimidazole is a group of anthelminthic drugs, which includes albendazole and mebendazole. They are shown to affect parasite locomotion and reproduction through action on the β-tubulin, compromising nematode’s cytoskeleton by impairing glucose uptake [33]. Albendazole is poorly absorbed and a single dose is shown to have an efficacy rate of 62.2% [33,34]. 

### 2.2. Macrocyclic Lactones (Ivermectin)

Macrocyclic lactones (MLs), in which ivermectin is the only approved drug for use in humans, act on nematodes residing in mammals’ gastrointestinal tract or lungs, inhibiting their capacity to move and feed, which results in their death [35]. Ivermectin is a very effective drug against early and adult stages of gastrointestinal parasites, and less effective against adult stages of filarial nematodes [35]. Macrocyclic lactones including ivermectin are known to react with a range of ligand-gated ion channels (α7 nACh receptors, acetylcholine-gated chloride channels, GABA-gated chloride channels, histamine-gated chloride channels, glycine receptors, and P2X4 receptors). The anthelminthic activity is shown by ivermectin interacting with glutamate-gated chloride channels (GluCl) in nematodes, increasing chloride permeability, which results in nematode paralysis [36,37]. Ivermectin is currently the best treatment for onchocerciasis and administered at intervals of one year in highly prevalent countries. While it is also effective at treating other helminth infections, it is not available in the onchocerciasis-free areas and recommended to be substituted with diethylcarbamazine [24,38]. Due to the strong protein binding ability of ivermectin, its oral administration can be impaired in strongyloidiasis disseminated patients. There is, however, no parenteral administration of ivermectin licensed currently, which is essential in cases of disseminated strongyloidiasis [39,40].

### 2.3. Anthelminthic Drugs Associated Issues

Treatment of soil-transmitted helminthiasis is challenging due to development of resistance, as demonstrated in veterinary practice, and reinfection occurrence [41,42]. Among soil-transmitted helminth infections, strongyloidiasis is the most difficult to treat because of its unique ability of autoinfection, especially in cases of hyperinfection or disseminated diseases [1,2,25,31,43]. The drug treatment efficacy depends also on number of factors including an individual’s immune system, co-infection with HTLV-1 and history of drug intake [44,45,46,47]. Fecal examination, traditionally used for monitoring treatment efficacy, is associated with low sensitivity. Although less available in low resource settings, serology tests are known for higher sensitivity and accuracy, and should be used for not only strongyloidiasis diagnosis but also follow-up tests [48,49].

The drugs, while reasonably well tolerated, can cause adverse effects including liver disfunction, gastrointestinal symptoms (nausea, vomiting, loose stool, abdominal distension or pain), chest tightness or pain, itching, fever, cough and wheezing, dizziness, and neurological effects [50,51,52,53]. Another issue with anthelminthic drugs is their teratogenicity potential in pregnant women who have a high risk of developing iron-deficiency anemia [54]. 

Animal-infecting nematode resistance development results in need for new anthelminthic drugs to be introduced to the market. Nematode resistance to different drugs has been widely studied and demonstrated frequently in animal studies [31,41,42,55,56,57,58]. Resistance to the benzimidazole class of drugs has been shown to be up to 97.7% and 71% in gastrointestinal nematodes parasitizing horse and sheep respectively [28,29]. Resistance (66.7%) to benzimidazoles has been also determined in sheep *Strongyloides* spp. [30]. The most recently introduced anthelminthic drug, ivermectin, has been shown to be the most successful in helminth infection treatment with less resistance development compared with the benzimidazole drugs. Nevertheless, resistance has been demonstrated in the last few years in gastrointestinal nematodes (29%), and sheep *Strongyloides* spp. (40%) [29]. Treatment of sheep parasites two times per year caused a rapid drug resistance development demonstrating that resistance can occur even in low frequency drug application [59]. This suggests that human-infecting nematodes are also likely, at some stage in the future, to become resistant to the available drugs. This is also induced by continuous use of a one drug family over the years, as in case of ivermectin against strongyloidiasis [60]. Studies on benzimidazole drugs against human nematodes have reported low efficacy of drug treatment, calling for great attention and warning for possible resistance development [61,62]. To date, human nematode studies with ivermectin have shown no resistance to the drug [50].

A little is understood in the mechanism of resistance development in *S. stercoralis* or other human parasites to anthelminthic drugs. Satoh et al. (1999) have found that *S. stercoralis* specific antibody, IgG4, is associated with both resistance to albendazole and elevated level of HLA-DRBI*0901, suggesting that patients should be tested for this antibody prior to drug treatment to check for their therapeutic effect on them. However, no other reports are available showing the association between increased level of IgG4 and resistance in a parasite [60]. A human immune system changes in response to strongyloidiasis infection, in particular T and B cells. The immune system has two responses to infective filariform larvae and host adapted larvae, which start autoinfection [44]. There are two mechanisms of ivermectin resistance identified so far: alteration of the membrane transport protein called P-glycoprotein, which is responsible for the drug delivery to the cell membrane, and alteration of the Cl channel receptor [58,63,64,65].

It is a risk for resistance development in response to large scale drug administration programs within the parasite control programs. The presence of a free-living stage of *S. stercoralis*, sexual reproduction, and relatively short lifespan and generation time could contribute to quicker drug resistance development in nematodes. Generally it is thought that if different drugs target and involve different receptors, their combined use will delay resistance development [66]. However, if resistance in two drugs involves same mechanism, combined drug treatment may be overlooked. ABC transporters have been shown to be involved in both ivermectin and albendazole resistance, which can potentially enhance the resistance development if both drugs are used for treatment [64]. It has been shown in some nematodes that ivermectin selects on β-tubulin, which is a primarily receptor for albendazole [66]. 

Although it is more difficult to study and confirm anthelminthic resistance in human parasites due to number of factors, the potential for resistance is mostly overlooked and should be more carefully examined in drug treatment application [67]. Notably, there are many gaps identified in our understanding of the pharmacology of anthelminthic drugs despite the fact that that millions of people around the world are treated by these drugs [35]. 

Mass drug administration (MDA) is the main clinical approach to controlling highly prevalent neglected tropical diseases. Ivermectin, along with benzimidazole drugs, have been shown to be effective against intestinal helminth and schistosome infections. Coadministration of different anthelminthic drugs allows integrating control programs for intestinal helminth infections, lymphatic filariasis and onchocerciasis with schistosomiasis and food-borne trematode infections. However, MDA could be associated with a higher risk for resistance development, as more people are given the drug more often, including those that are no carrying disease. More research is required to study the long-term effects of repeated drug doses [38,68].

## 3. Nematicides

Nematicides are used to control plant parasite nematodes, which are ubiquitous and globally cause costly yield loses in agriculture [32,69]. To date, there have been limited studies demonstrating nematicides use on non-plant nematodes, as they are mostly treated by anthelminthic drugs. However, the mode of their action and species’ similarity might allow using them on animal and human parasites. 

According to the Australian Pesticides and Veterinary Medicines Authority there are currently around 20 registered nematicides to use in Australia with the four active compounds fenamiphos, fluensulfone, oxamyl and carbofuran (Table 2) [70]. The active constituents of used nematicides are of organophosphorus, carbamate and thiazole chemical groups.

### 3.1. Organophosphorus and Carbamate Nematicides (Fenamiphos, Oxamyl and Carbofuran) Mode of Action

Organophosphates and carbamates are non-fumigant nematicides. Organophosphorus and carbamate nematicides (fenamiphos, oxamyl and carbofuran) cause the paralysis of nematodes through inhibition of cholinesterase enzymes, which are responsible for acetylcholine neurotransmitter breakdown. Organophosphates and carbamates cause either irreversible or reversible inhibition of a cholinesterase enzyme blocking its function [71].

Not much research has been done on human parasitic nematodes including *S. stercoralis*; however, in *C. elegans*, acetylcholine is the neurotransmitter that controls nematode’s movement, pharyngeal pumping, and egg laying. When acetylcholinesterase/cholinesterase suppressed, acetylcholine builds up, transmitting nerve impulses and causing constant muscle and nerve contraction leading to the nematode’s exhaustion and tetany [72]. Oxamyl is known as a more effective nematicide than fenamiphos [73].

### 3.2. Thiazole (Fluensulfone) Mode of Action

Fluensulfone, a fluoroalkenyl thioether group drug, has different mode of action and effect on nematodes from those of organophosphorus and carbamate nematicides and also anthelminthic drugs such as ivermectin [74]. There have not been studies done describing its mode of action on nematodes. However, fluensulfone has shown to be highly effective against a number of plant nematodes [18,74]. In their study, Kearn et al. (2014) have studied fluensulfone effect on *C. elegans*, a genetic nematode model to study effects of different anthelminthic drugs and nematicides that are used against animal and human parasites [32]. It has been shown that a slightly higher dose of fluensulfone is required to have a similar effect on *C. elegans* as on plant parasite nematodes, inhibiting egg laying, hatching, development, feeding and moving stages of the nematode [74]. 

### 3.3. Nematicides Associated Issues (Toxic Effects and Resistance to Nematicides)

Most cholinesterase inhibiting nematicides have been banned or restricted for use due to their adverse toxic effects on non-target organisms including humans, and the environment, which is associated with absence of species’ selectivity [32,71]. Another disadvantage of non-fumigant nematicides is their mobility in soil which can potentially cause widespread non-target toxic effects. Oxamyl and fenamiphos are known for leaching from the site of application [75,76]. Carbofuran has been banned for use in European Union in 2009 (Regulation 1107/2009), Canada and U.S. due to its adverse side-effects [77].

A study on fenamiphos, oxamyl and carbofuran effects on *C. elegans* has shown AChE recovery ability by nematodes in response to all the three nematicides. It has been also shown that only small recovery of the enzyme is required for nematode moving restoration and normal behaviour [78].

There are currently no studies available on the non-specific toxicity of fluensulfone. However, the acute LD_50_ value for rats via for oral administration of fluensulfone is much lower compared with organophosphate nematicides [18,79]. 

It is commonly thought that nematicide resistance for plant nematodes is not as great a concern as for animal nematodes, hence there are limited studies exploring potential plant nematodes’ resistance compared to the numerous studies on animal nematodes’ resistance. It is thought that there is a lesser potential for the development of plant nematicide resistance due to number of factors. These include: nematicides altering the selection pressure on plant parasitic nematodes, mitotic parthenogenesis in plant nematodes leading to less genetic diversity, and biodegradation of nematicides by soil bacteria [80]. However, these factors can probably delay but not prevent resistance development. It is known that resistance is more likely to develop with persistent compounds such as organophosphorus and carbamate substances rather than short-lived molecules [81]. Plant nematodes have been shown to be quite adaptive to chemical treatment. *Rhabditis oxycerca, Criconemella xenoplax, Xiphinema index, Meloidogyne incognita and Pratylenchus vulnus* have developed high resistance to organophosphates and carbomates after long-term exposure [82,83].

## 4. Conclusions

While nematicides are extensively used against plant nematodes, their use is limited or non-existent in human parasite control. An overlooked environmental approach in strongyloidiasis control is to kill free-living parasites in environment before they get into a human host.

Above we have assessed commercially available drugs used to treat strongyloidiasis and explored the main issues associated with these drug treatments. This includes the emergence of drug resistance in numerous animal nematodes when applied in veterinary practice. This highlights the potential for resistance in human helminths, which is a particular problem for *S. stercoralis* as currently there is only two drugs approved for human treatment. Other issues with treatment include drugs’ inability to prevent reinfection, and potential for problems associated with drug administration during pregnancy. Nematicides have potential to be used on free-living *Strongyloides* nematodes. A combined approach to fight strongyloidiasis should consider environmental control as well as drug treatment. Future studies could consider focusing initial efforts on the nematicide fluensulfone, which has been shown to have the least toxic effect on the environment and non-target species, and desirable effects on all the stages of a nematode, as demonstrated by a model parasite, *C. elegans*.

## Figures and Tables

**Table 1:** WHO recommended anthelminthic drugs to treat strongyloidiasis.

#	Drug Name	Class (Drench Group)	Mode of Action *	Resistance of Gastrointestinal Nematodes (Veterinary Studies)	Resistance of *Strongyloides* spp. (Veterinary Studies)
1a	Albendazole	Benzimidazole, BZ, “white” (introduced in 1961)	Interaction with β-tubulin impairing cytoskeleton	1. Horse (97.7%) [28] 2. Sheep (71%) [29]	1. Sheep (57%)[29], Sheep (66.7%)[30] 2. Horse [31]
1b	Mebendazole				
2	Ivermectin	Macrocyclic lactone, ML, “ectin” (introduced in 1980s)	Paralysis of pharyngeal and body wall musculature	1. Sheep (29%) [29]	1. Sheep (43%) [29]

* Source: [32].

**Table 2:** Registered in Australia nematicides and their active constituents.

#	Active constituent	Chemical group	No. of registered nematicides	Mode of action
1	Fenamiphos	Organophosphorus	14	Inhibition of cholinesterase
2	Oxamyl	Carbamate-methylcarbamate	1	
3	Carbofuran		2	
4	Fluensulfone	Thiazole	1	
